# Clinical Characteristics, Comorbidities, and Response to Treatment of Veterans With Obstructive Sleep Apnea, Cincinnati Veterans Affairs Medical Center, 2005-2007

**DOI:** 10.5888/pcd9.110117

**Published:** 2012-01-26

**Authors:** Pamela Samson, Kenneth R. Casey, James Knepler, Ralph J. Panos

**Affiliations:** Washington University in St. Louis School of Medicine, St. Louis, Missouri. At the time of this study, Ms Samson was affiliated with the Cincinnati Veterans Affairs Medical Center, Cincinnati, Ohio; Cincinnati Veterans Affairs Medical Center, Cincinnati, Ohio; University of Arizona, Tucson, Arizona. At the time of this study, Knepler was affiliated with the Cincinnati Veterans Affairs Medical Center, Cincinnati, Ohio; Pulmonary, Critical Care, and Sleep Division, Cincinnati Veterans Affairs Medical Center, Cincinnati

## Abstract

**Introduction:**

Obstructive sleep apnea (OSA) is a common disorder that is associated with significant morbidity. Veterans may be at an elevated risk for OSA because of increased prevalence of factors associated with the development and progression of OSA. The objective of this study was to determine the clinical characteristics, comorbidities, polysomnographic findings, and response to treatment of veterans with OSA.

**Methods:**

We performed a retrospective chart review of 596 patients undergoing polysomnography at the Cincinnati Veterans Affairs Medical Center from February 2005 through December 2007. We assessed potential correlations of clinical data with polysomnography findings and response to treatment.

**Results:**

Polysomnography demonstrated OSA in 76% of patients; 30% had mild OSA, 23% moderate OSA, and 47% severe OSA. Increasing body mass index, neck circumference, Epworth Sleepiness Scale score, hypertension, congestive heart failure, and type 2 diabetes correlated with increasing OSA severity. Positive airway pressure treatment was initiated in 81% of veterans with OSA, but only 59% reported good adherence to this treatment method. Of the patients reporting good adherence, a greater proportion of those with severe OSA (27%) than with mild or moderate disease (0%-12%) reported an excellent response to treatment.

**Conclusion:**

The prevalence of metabolic and cardiovascular comorbidities increased with increasing OSA severity. Only 59% of treated patients reported good adherence to treatment with positive airway pressure, and response to treatment correlated with OSA severity.

## Introduction

Obstructive sleep apnea (OSA), a condition characterized by repeatedly interrupted breathing during sleep, occurs frequently in adults (1). The prevalence of OSA increases with age and may affect 38% to 68% of people older than 60 years (1). Clinical characteristics that predict risk of development and progression of OSA include a large neck circumference and male sex. Body mass index (BMI) and tonsil size are predictors of OSA severity (2,3). Comorbid conditions associated with OSA include hypertension, atrial fibrillation, congestive heart failure, stroke, metabolic syndrome, and type 2 diabetes (2,4,5). Patients cared for by the Veterans Health Administration (VHA) are predominantly older men with many of these conditions (6). A survey of veterans in northeast Ohio using the Cleveland Sleep Habits questionnaire (7) showed that 46% of the respondents were at high risk for OSA (7). A similar study in San Juan, Puerto Rico, showed that 34% of veterans attending ambulatory clinics were at high risk for OSA (8).

OSA is diagnosed by polysomnography and measured by the apnea-hypopnea index (AHI). An AHI of more than 5 events per hour (9) is diagnosed as OSA. OSA severity is stratified according to AHI score. Fewer than 5 events per hour is designated as normal, 5 to 14 events per hour as mild OSA, 15 to 30 events per hour as moderate OSA, and more than 30 events per hour as severe OSA (9). Once OSA is diagnosed, a continuous positive airway pressure (CPAP) study is often performed to determine the optimal positive airway pressure required to reduce the AHI and improve oxygenation. The most common treatment for OSA, positive airway pressure (PAP) treatment, is frequently initiated to reduce sleep-related symptoms. Patients with more sleep-related symptoms appear to receive greater benefit from treatment than do patients with fewer sleep-related symptoms (10). Despite the availability of numerous types of masks and interfaces, CPAP is often poorly tolerated, and it is difficult to predict which patients will adhere and respond to treatment (11). The objective of this study was to determine the clinical characteristics, comorbidities, polysomnographic findings, and response to therapy of veterans with OSA.

## Methods

We reviewed the records of 596 patients who underwent polysomnography during 3 years at the Cincinnati Veteran Affairs Medical Center (VAMC). Patients were evaluated on the basis of their AHI, OSA severity, clinical characteristics (eg, neck circumference, BMI), comorbidities, and response to treatment. This protocol was approved by the research and development committee of the Cincinnati VAMC and reviewed by the University of Cincinnati institutional review board, which waived the need for consent.

### Participant selection

Health care providers throughout the Cincinnati VAMC referred veterans with suspected sleep disorders to our sleep clinic, where a standardized sleep evaluation was performed and polysomnography scheduled. We retrospectively reviewed the medical records and polysomnography reports of 748 veterans who completed evaluation and testing for sleep-disordered breathing in the Cincinnati VAMC from February 2005 through December 2007. From this chart review, we selected for our study group 596 patients who completed the evaluation and polysomnography testing. We excluded 152 patients with previously diagnosed OSA who returned for therapeutic (CPAP/bilevel titration) studies and patients who did not complete testing, terminated the test prematurely, or achieved insufficient or no sleep. All patients who were referred for polysomnography completed a pretest assessment and questionnaire with assistance from the sleep study technologist. Information abstracted from this questionnaire included age, measured weight, self-reported height, smoking history, Epworth Sleepiness Scale (ESS) score (a measure of sleep propensity) (12), and self-reported snoring, apneas, and morning headaches.

We used a Sandman Elite sleep system for polysomnography studies (Sandman Elite, version 8.0, Nellcor Puritan Bennett [Melville] Ltd, Kanata, Ontario, Canada). Monitored channels included bilateral oculograms, 4 electroencephologram channels, electrocardiogram, bilateral anterior tibialis electromyograms (EMG), chin EMG, body position, video channel, PAP level and flow, and snoring microphone. Nasal/oral and PAP airflow were measured by thermocouple, thoracic and abdominal respiratory effort by piezoelectric method, and oxygen saturation (SpO_2_) by pulse oximetry. We analyzed and scored data according to criteria of the American Academy of Sleep Medicine (9). We defined apnea as cessation of airflow for at least 10 seconds and hypopnea as a reduction in airflow of at least 30% lasting at least 10 seconds, accompanied by at least a 4% decrease in oxygen saturation (9). Rapid eye movement (REM) rebound was defined as 20% or more of sleep time in REM. In many of our polysomnography studies we used a split-night protocol consisting of an initial diagnostic study followed by titration of CPAP or bilevel treatment on the same night. (CPAP maintains a constant minimal airway pressure throughout the respiratory cycle whereas bilevel treatment oscillates between a higher inspiratory pressure to maintain airway patency and a lower expiratory pressure to facilitate exhalation. Both are forms of positive airway pressure.) Patients with OSA who did not complete a split-night protocol because of an insufficient number of events, too little sleep, or too little REM sleep during the first half of the night returned for a titration study on another night.

Abstracted polysomnography data included total sleep time; sleep latency; REM latency; percentage of sleep achieved in stages 1, 2, 3-4, and REM; number of central, obstructive, and mixed apneas; number of hypopneas; REM-related AHI; and minimal SpO_2_. If treatment was initiated, we reviewed these same values as well as AHI at optimal treatment pressure. We obtained patient medical history and information on comorbid conditions (ie, hypertension, coronary artery disease, congestive heart failure, atrial fibrillation, pulmonary hypertension, type 2 diabetes, cardiovascular accidents, and transient ischemic attacks) from the Cincinnati VAMC electronic medical record. We reviewed all clinical reports from postpolysomnography encounters to assess the patient's adherence to treatment and response to therapy. We graded adherence according to the following criteria: "good," patient reported use of positive pressure equipment for 3 or more nights weekly; "partial," patient reported use of equipment for fewer than 3 nights weekly; "not adherent," no use of equipment; and "not specified/no data," patient had not returned to the sleep clinic for follow-up or there were no comments regarding adherence in other clinical notes. For veterans with good adherence, we graded the response to treatment according to the following criteria: "excellent, "complete or near complete relief of pretreatment sleep-related symptoms, greatly improved energy and alertness, and more restful sleep; "moderate," relief of most sleep-related symptoms but persistent daytime somnolence or fatigue and inconsistently restorative sleep; "no change," persistence of nearly all sleep-related symptoms; and "not specified/no data," patients had not returned to the sleep clinic for follow-up or there were no comments regarding sleep-disordered breathing in records of other clinical encounters.

Because of the high prevalence of severe OSA, we performed further comparisons to determine whether patients with ultrasevere OSA (AHI >60 events/h, 1 respiratory event/min) could be distinguished from those with less severe OSA (AHI 31-60 events/h).

### Statistical analysis

We calculated mean, standard deviation (SD), standard error of the mean, and confidence intervals for continuous variables. Differences between the categorical OSA groups and continuous variables were analyzed by using 1-way ANOVA with the Bonferroni test for multiple comparisons. We calculated categorical variables as frequencies or proportions and analyzed them using χ^2^ testing with the Marascuilo procedure for multiple comparisons. We defined significant differences as *P* < .05. We performed all statistical analyses with GraphPad Prism 5.0 statistical software (GraphPad, La Jolla, California).

## Results

Patients were predominantly male (559 of 596 [94%]), with a mean (SD) age of 56.0 (11.6) years. Polysomnography demonstrated OSA in 76% of patients; 30% had mild OSA, 23% moderate OSA, and 47% severe OSA. Increasing BMI, neck circumference, ESS scores, hypertension, congestive heart failure, and type 2 diabetes correlated with increasing OSA severity (Table 1).

Among the OSA patients, the REM-related AHI rose and the SpO_2_ declined as OSA severity increased (Table 2).

Treatment was initiated for 81% of the patients with OSA; 73% of patients received CPAP and 27% received bilevel therapy. With CPAP, the proportion of patients with REM rebound increased with increasing OSA severity; one-third of patients with severe OSA experienced REM rebound (Table 2). More than 10% of patients did not tolerate CPAP (pulled off the mask during the study or requested removal of the mask), and treatment adherence did not vary with OSA severity. The AHI declined dramatically with successful CPAP for all patients with OSA, and the posttreatment AHI was lower in the mild group compared with the severe group. The optimal levels of CPAP and inspiratory and expiratory bilevel pressures rose with increasing OSA severity.

### Adherence and outcomes

Follow-up information about adherence to treatment was available for 291 of the 368 treated patients (79%). Of the 291, 172 patients (59%) reported using their CPAP or bilevel equipment at least 3 nights weekly, and 27 of 100 (27%) patients with severe OSA reported an excellent response compared with 0 of 40 patients with mild OSA (Table 3).

### Ultrasevere and less severe OSA

Patients with more than 30 AHI events per hour (n = 211) were divided into less severe (n = 99) and ultrasevere (n = 112) categories. More patients with ultrasevere OSA reported a history of observed apnea events, a higher BMI, and concurrent coronary artery disease and pulmonary hypertension than did patients with less severe OSA. Although the minimal SpO_2_ was less in the ultrasevere group, other polysomnographic findings, treatments, adherence, and outcomes were similar in the 2 groups.

## Discussion

In our study group of 596 patients who underwent complete diagnostic polysomnography testing, 76% had OSA. Of these, 30% had mild, 23% moderate, and 47% severe OSA. BMI, neck circumference, and ESS score increased with worsening OSA severity, as did cardiovascular and metabolic comorbidities. Most patients were treated for OSA, but only 59% reported good adherence with positive pressure therapy. More adherent patients with severe OSA than with mild or moderate disease reported an excellent response to treatment. Finally, despite a higher proportion of patients with severe OSA, we were unable to determine clinical or polysomnographic features that distinguished less severe OSA from ultrasevere OSA.

Previous studies within the VHA have shown that 34% to 47% of veterans attending outpatient clinics are at increased risk for OSA (7,8). In 1983, a preliminary study of 27 randomly selected inpatients at the San Diego VAMC who underwent portable polysomnography monitoring of 4 channels (thoracic and abdominal respiratory effort, lower-extremity electromyogram, and wrist actigraphy) in their hospital beds demonstrated that 7 (27%) had sleep apnea, defined as 30 or more apneas per hour (13). Subsequent studies at the same institution using the same study protocol found that 84% of 436 randomly selected inpatients had an AHI greater than 5 in 1991, and 53% of 186 inpatients had an AHI greater than 15 in 2003 (14,15). In contrast, a review of the first 117 patients undergoing polysomnography by the same group in the San Diego Sleep Disorders Clinic showed that 44% had sleep apnea (16). Approximately one-fourth (46 of 192) of Persian Gulf War veterans self-referred to the Comprehensive Clinical Evaluation Program at Fort Sam Houston had histories suggesting a sleep disorder; polysomnography demonstrated OSA (defined as a respiratory disturbance index of ≥15 events/h) in 33% of these veterans (17). Differences in technology, study protocols including the tested population, and definition of OSA make it difficult to compare these reports with our study, which demonstrated OSA (AHI ≥5 events/h) in 76% of veterans undergoing polysomnography, 47% of whom had severe OSA (AHI >30 events/h).

Based on previous estimates of the proportion of the veteran population that is at increased risk of OSA (34%-47%) (7,8) and our polysomnography results (76% with demonstrated OSA, 47% of whom had severe OSA), approximately 26% to 36% of veterans served by the VHA would be diagnosed with OSA, and 12% to 17% would have severe OSA if all veterans at increased risk for sleep disordered breathing completed diagnostic polysomnography testing. In a review of VHA administrative databases from 1998 to 2001, Sharafkhaneh and colleagues (18) found that the prevalence of coded and documented diagnosed OSA was 2.9%. Thus, sleep apnea may be underrecognized and underdiagnosed in veterans receiving care in the VHA system, and possibly only 1 of every 5 to 10 veterans with OSA is diagnosed. Prospective, multicenter epidemiologic studies are needed to determine the precise prevalence and severity of OSA among veterans served by the VHA.

Previous population-based studies suggest that 15% to 32% of men in the general American population have OSA and that the prevalence of severe OSA is approximately 5% (1). These prevalence calculations are very similar to our estimated prevalence of OSA and severe OSA in the national veteran population, 26% to 36% and 12% to 17%, respectively. These national studies include people aged 20 to 99 and, since the prevalence of OSA appears to begin to increase with age in midlife, may not be comparable to the national veteran population (1). Furthermore, the veteran population may have a higher prevalence of factors associated with the development and progression of OSA, such as excess body weight, smoking, alcohol consumption, and nasal congestion (1). Thus, comparison of the veteran population with an age-, sex-, and risk-factor-matched cohort from the general American population is required to determine whether the prevalence and severity of OSA are the same in both groups.

In our study, BMI, neck circumference, and ESS score correlated positively with AHI. Participants in the Sleep Heart Health Study (SHHS) who had an AHI of 15 or more were significantly more likely to have an increased BMI, neck circumference, and breathing-pause frequency (2). The SHHS did find a correlation between habitual snoring and loud snoring and AHI of 15 or more, which we did not see in our study (2). Of all the patient attributes evaluated in the SHHS, self-reported, frequent apneas (>3 nights/wk) occurred most frequently among those with an AHI of 15 or more (49%), but this finding alone was only minimally predictive of OSA (2). BMI and neck circumference are strong predictors of OSA, whereas self-reported apneas, ESS values, and frequent, loud snoring predict OSA severity (2,19).

In 118,105 veterans diagnosed with OSA, metabolic and cardiovascular comorbidities occurred frequently: diabetes in 32.9%, obesity in 30.5%, hypertension in 60.1%, cardiovascular disease in 27.6%, congestive heart failure in 13.5%, and cerebrovascular accident in 5.7% (17). In our study, the prevalence of hypertension, congestive heart failure, and type 2 diabetes correlated with OSA severity. Large studies have shown a positive association between hypertension and OSA severity (1,20). In a study of nearly 2,300 people in China undergoing polysomnography, AHI was linearly related to systolic and diastolic blood pressure up to an AHI of 60 (19). Others have also shown that the prevalence of diabetes increases with the severity of OSA (21).

The minimal measured SpO_2_ declined with increasing OSA severity. Various indices of nocturnal oxygen saturation have been shown to correlate with and predict AHI (22,23). Lin and colleagues (23) showed that the oxyhemoglobin desaturation index was the most sensitive and specific measure of oxygenation for all levels of OSA.

For many patients, apneas and hypopneas can be more prominent during REM sleep (24). A Japanese study found that patients with an AHI of 60 or more were significantly more likely to have a higher AHI in non-REM sleep than in REM sleep, whereas among patients with less severe disease, the relationship was reversed (25). Another investigation showed that half of patients with OSA have a higher non-REM AHI than REM AHI (26). The REM-related AHI correlated with AHI and increased most dramatically when AHI was greater than 60 events per hour.

Our study showed that patients with severe OSA were slightly more likely to adhere to CPAP treatment, a finding similar to that of other investigations (27,28). In our study, 53% of patients with severe OSA had good adherence to treatment, whereas only 39% of those with mild OSA reported using their equipment more than 3 nights weekly. Adherence to CPAP use is better in people with more daytime sleepiness regardless of OSA severity (10,29). The ESS score, a measure of excessive daytime sleepiness, was significantly higher in patients with more severe OSA, suggesting that these patients were more symptomatic and may have experienced more symptom improvement with treatment. The higher proportion of excellent response to treatment among Cincinnati VMAC patients with severe OSA corresponds to results of previous studies that found significant associations between the resolution of symptoms with CPAP treatment and improved treatment adherence (30,31).

This study was a retrospective review of polysomnography studies at a single center, the Cincinnati VAMC sleep center. Patients with more severe sleep-related symptoms may have been preferentially referred for sleep evaluation, resulting in higher prevalence and severity of OSA. Only completed diagnostic polysomnography studies were analyzed; including patients who did not complete testing and may not have had OSA would reduce the OSA diagnosis rate. In most of the patients we studied, we used a split-night polysomnography protocol that may have underestimated the presence and severity of OSA. Another limitation was the use of self-reporting for adherence assessment. Although patients' CPAP and bilevel units were examined for the numbers of hours used per night, this evaluation was not performed consistently, and there were insufficient data for analysis. Finally, the severity of hypertension and treatment for hypertension at the time of the polysomnography study were not documented. Only the presence or absence of a hypertension diagnosis was noted.

On the basis of our data and on previous surveys of the prevalence of patients at high risk for OSA within the VHA, we estimate the prevalence of OSA to be 26% to 36% of veterans cared for by the VHA, and the prevalence of severe OSA to be 12% to 17%. Metabolic and cardiovascular comorbidities occurred frequently in veterans with OSA, and the prevalence of these disorders increased with OSA severity. Only 59% of treated patients at the Cincinnati VAMC reported good adherence with CPAP treatment, and within this group, response to therapy increased as OSA severity worsened.

## Figures and Tables

**Table 1:** Clinical Characteristics of Veterans (N = 596) With Obstructive Sleep Apnea, Cincinnati Veterans Affairs Medical Center, 2005-2007

Characteristic	Obstructive Sleep Apnea Severity^a^				*P* Value^b^

	None (n = 144)	Mild (n = 136)	Moderate (n = 105)	Severe(n = 211)	
**Age, mean (SD), y**	54.0 (13.1)	55.6 (11.9)	56.9 (9.4)	57.1 (11.3)	.07
**Male sex, n (%)**	124 (86.1)	128 (94.1)	101 (96.2)	206 (97.6)	<.001^c^
**Health history**					
Morning headaches, n (%)	52 (38.9)	52 (30.4)	41 (44.6)	75 (39.7)	.69
Epworth Sleepiness Scale^d^, mean (SD)	11.5 (5.7)	12.2 (5.1)	11.7 (5.8)	14.0 (5.4)	<.001^e^
Self-reported snoring, n (%) (n = 535)^f^	122 (99.0)	122 (100)	93 (98.9)	192 (98.0)	.19
Self-reported apneas, n (%) (n = 413)^f^	78 (87.6)	86 (95.5)	71 (87.6)	153 (93.9)	.09
**Physical examination**					
BMI, mean (SD), kg/m^2^	31.3 (5.8)	34.7 (7.2)	35.9 (7.4)	37.4 (8.5)	<.001^e^
Neck circumference, mean (SD), in	16.9 (1.6)	17.9 (1.7)	17.7 (1.8)	18.1 (1.6)	<.001^e^
**Comorbidities**					
Hypertension, n (%)	89 (61.8)	106 (77.9)	81 (77.1)	172 (81.5)	<.001^c^
Coronary artery disease, n (%)	34 (23.6)	36 (26.5)	27 (25.7)	55 (26.1)	.38
Congestive heart failure, n (%)	13 (9.0)	14 (10.3)	6 (5.7)	33 (15.6)	.04
Atrial fibrillation, n (%)	5 (3.5)	9 (6.6)	5 (4.8)	10 (4.7)	.82
Pulmonary hypertension, n (%)	3 (2.1)	6 (4.4)	3 (2.9)	6 (2.8)	.40
Type 2 diabetes, n (%)	33 (22.9)	69 (50.7)	44 (41.9)	98 (46.4)	<.001^g^
Cardiovascular accidents, n (%)	9 (6.3)	7 (5.1)	5 (4.8)	15 (7.1)	.43
Transient ischemic attacks, n (%)	1 (0.1)	3 (2.2)	0	2 (0.9)	.67
**Smoking history**					
Current smoker, n (%)	54 (37.5)	30 (22.0)	39 (37.1)	62 (29.3)	.02
Never smoked, n (%)	20 (13.9)	24 (17.6)	20 (19.0)	46 (21.8)	.09

Abbreviation: SD, standard deviation; BMI, body mass index.

a None, apnea-hypopnea index (AHI) <5; mild, AHI 5-14; moderate, AHI 15-30; severe, AHI >30.

b ANOVA with Bonferonni correction was used to compare continuous values and χ^2^ test with Marasculio procedure was used to compare proportional variables.

c None vs severe.

d Johns (12).

e None vs severe, mild vs severe, moderate vs severe.

f Data were not available for all patients; n = number of patients with this information.

g None vs mild, moderate, and severe.

**Table 2:** Polysomnographic Findings and Treatment of Patients With Obstructive Sleep Apnea (N = 596), Cincinnati Veterans Affairs Medical Center, 2005-2007

Findings/Treatment	Obstructive Sleep Apnea Severity^a^				*P* Value^b^

	None (n = 144)	Mild (n = 136)	Moderate (n = 105)	Severe (n = 211)	
**Polysomnographic findings**					
Pretreatment AHI, mean (SD), events/h	1.5 (1.9)	9.2 (2.9)	21.3 (4.3)	66.9 (27.5)	NA
Pretreatment REM-related AHI, mean (SD), events/h	5.5 (13.2)	29.7 (22.9)	44.0 (32)	54.1 (34.5)	<.001^c^
Minimum SpO_2_, mean (SD), %	88.4 (4.5)	83.4 (6.3)	81.9 (8.1)	78.4 (9.3)	<.001^d^
**Treatment**					
Patients receiving CPAP treatment, n (%)	NA^e^	82 (60.3)	56 (53.3)	129 (61.1)	.60
CPAP pressure, mean (SD), cm H_2_O	NA^e^	8.2 (2.3)	8.3 (1.9)	9.9 (2.5)	<.001^d^
Patients receiving bilevel treatment, n (%)	NA^e^	21 (15.4)	21 (20.0)	59 (28.0)	.02^f^
Bilevel pressure inspiration, mean (SD), cm H_2_O	NA^e^	12.0 (2.5)	13.3 (2.7)	14.5(3.2)	.002^f^
Bilevel pressure expiration, mean (SD), cm H_2_O	NA^e^	7.8 (2.5)	9.2 (2.6)	10.2 (3.0)	.003^f^
Did not tolerate CPAP or bilevel treatment, n (%)	NA^e^	13 (9.6)	17 (16.2)	17 (8.0)	.08
REM rebound, n (%) (n = 435)^g^	NA^e^	12 (8.8)	21 (20.0)	64 (33.0)	<.001^f^
Posttreatment AHI, mean (SD), events/h	NA^e^	3.0 (5.1)	3.6 (4.7)	5.6 (9.5)	.04^f^

Abbreviations: AHI, apnea-hypopnea index; SD, standard deviation; NA, not applicable; REM, rapid eye movement; SpO_2_, pulse oximetry oxygen saturation; CPAP, continuous positive airway pressure.

a None, AHI <5; mild, AHI 5-14; moderate, AHI 15-30; severe, AHI >30.

b ANOVA with Bonferonni correction was used to compare continuous values, and χ^2^ test with Marasculio procedure was used to compare proportional variables.

c Mild vs moderate and severe.

d Mild vs severe, moderate vs severe.

e Treatment data are only for patients with OSA.

f Mild vs severe.

g REM rebound was defined as 20% of sleep time in REM; no. is the number of patients with data concerning REM rebound. Data were not available for all patients; n = number of patients with this information.

**Table 3:** Adherence of Patients With Obstructive Sleep Apnea (n = 368) Treated With Positive Airway Pressure and Response in Patients with Good Adherence to Treatment (n = 172), Cincinnati Veterans Affairs Medical Center, 2005-2007

Adherence/Response	Obstructive Sleep Apnea Severity,^a^ n %			*P* Value^b^

	Mild (n = 103)	Moderate (n = 77)	Severe (n = 188)	
**Adherence^c^**				
Good	40 (39)	32 (42)	100 (53)	.09
Partial	20 (19)	12 (16)	21 (11)	
Not adherent	18 (17)	12 (16)	36 (19)	
Not specified/no data	25 (24)	21 (27)	31 (16)	
**Response^d^**				
Excellent	0	4 (12)	27 (27)	.01^e^
Moderate	25 (62)	17 (53)	49 (49)	
No change	1 (2)	0	2 (2)	
Not specified/no data	14 (35)	11 (34)	22 (22)	

a None, AHI <5; mild, AHI 5-14; moderate, AHI 15-30; severe, AHI >30.

b  χ^2^ test with Marasculio procedure was used to compare proportional variables.

c Good, self-reported use of positive pressure equipment for ≥3 nights weekly; partial, self-reported use of equipment <3 nights weekly; not adherent, no use of equipment; not specified/no data, patient did not return to the sleep clinic for follow-up or there were no comments regarding adherence in other clinical notes.

d Excellent, complete or near complete relief of pretreatment sleep-related symptoms, greatly improved energy and alertness, and more restful sleep; moderate, relief of most sleep-related symptoms but persistent daytime somnolence or fatigue and inconsistently restorative sleep; no change, persistence of nearly all sleep-related symptoms; not specified/no data, patients did not return to the sleep clinic for follow-up or there were no comments regarding sleep disordered breathing in other clinical notes.

e Mild vs severe.

## References

[1] Young T, Peppard PE, Gottlieb DJ (2002). Epidemiology of obstructive sleep apnea a population health perspective. Am J Respir Crit Care Med.

[2] Young T, Shahar E, Nieto FJ, Redline S, Newman AB, Gottlieb DJ (2002). Predictors of sleep-disordered breathing in community-dwelling adults: the Sleep Heart Health Study. Arch Intern Med.

[3] Erdamar B, Suoglu Y, Cuhadaroglu C, Katircioglu S, Guven M (2001). Evaluation of clinical parameters in patients with obstructive sleep apnea and possible correlation with the severity of the disease. Eur Arch Otorhinolaryngol.

[4] Young T, Evans L, Finn L, Palta M (1997). Estimation of the clinically diagnosed proportion of sleep apnea syndrome in middle-aged men and women. Sleep.

[5] Peled N, Kassirer M, Shitrit D, Kogan Y, Shlomi D, Berliner AS, Kramer MR (2007). The association of OSA with insulin resistance, inflammation and metabolic syndrome. Respir Med.

[6] Ashton CM, Petersen NJ, Wray NP, Yu HJ (1998). The Veterans Affairs medical care system: hospital and clinic utilization statistics for 1994. Med Care.

[7] Mustafa M, Erokwu N, Ebose I, Strohl K (2005). Sleep problems and the risk for sleep disorders in an outpatient veteran population. Sleep Breath.

[8] Ocasió-Tascon ME, Alicea-Colón E, Torres-Palacios A, Rodriquez-Cintrón W (2006). The veteran population: one at high risk for sleep-disordered breathing. Sleep Breath.

[9] Epstein LJ, Kristo D, Strollo PJ, Friedman N, Malhotra A, Patil SP (2009). Clinical guideline for the evaluation, management and long-term care of obstructive sleep apnea in adults. J Clin Sleep Med.

[10] Patel SR, White DP, Malhotra A, Stanchina ML, Ayas NT (2003). Continuous positive airway pressure therapy for treating sleepiness in a diverse population with obstructive sleep apnea: results of a meta-analysis. Arch Intern Med.

[11] Collen J, Lettieri C, Kelly W, Roop S (2009). Clinical and polysomnographic predictors of short-term continuous positive airway pressure compliance. Chest.

[12] Johns MW (1991). A new method for measuring daytime sleepiness: the Epworth Sleepiness Scale. Sleep.

[13] Kreis P, Kripke DF, Ancoli-Israel S (1983). Sleep apnea: a prospective study. West J Med.

[14] Ancoli-Israel S, Kripke DF (1991). Prevalent sleep problems in the aged. Biofeedback Self Regul.

[15] DuHamel ER, Stepnowsky C, Engler R, Cohen-Zion M, Marler M (2003). The relationship between congestive heart failure, sleep apnea, and mortality in older men. Chest.

[16] Ancoli-Israel S, Kripke DF, Menn SJ, Messin S (1981). Benefits of a sleep disorders clinic in a Veterans Administration Medical Center. West J Med.

[17] Peacock MD, Morris MJ, Houghland MA, Anders GT, Blanton HM (1997). Sleep apnea-hypopnea syndrome in a sample of veterans of the Persian Gulf War. Mil Med.

[18] Sharafkhaneh A, Richardson P, Hirschkowitz M (2004). Sleep apnea in a high risk population: a study of Veterans Health Administration beneficiaries. Sleep Med.

[19] Dixon JB, Schachter LM, O'Brien PE (2003). Predicting sleep apnea and excessive day sleepiness in the severely obese: indicators for polysomnography. Chest.

[20] He QY, Feng J, Zhang XL, Liang ZA, Huang SG, Kang J (2010). Relationship of daytime blood pressure and severity of obstructive sleep apnea among Chinese: a multi-center investigation in China. Chin Med J (Engl).

[21] Ronksley PE, Hemmelgarn BR, Heitman SJ, Hanly PJ, Faris PD, Quan H, Tsai WH (2009). Obstructive sleep apnoea is associated with diabetes in sleepy subjects. Thorax.

[22] Magalang UJ, Dmochowski J, Veeramachaneni S, Draw A, Mador MJ, El-Solh A, Grant BJ (2003). Prediction of the apnea-hypopnea index from overnight pulse oximetry. Chest.

[23] Lin CL Yeh C Yen CW Hsu WH Hang LW 135 1 2009 86 93 Comparison of the indices of oxyhemoglobin saturation by pulse oximetry in obstructive sleep apnea hypopnea syndrome Chest Li 18689584 10.1378/chest.08-0057

[24] Findley LJ, Wilhoit SC, Suratt PM (1985). Apnea duration and hypoxemia during REM sleep in patients with obstructive sleep apnea. Chest.

[25] Muraki M Kitaguchi S Ichihashi H Haraguchi R Iwanage T Kubo H 36 5 2008 906 913 Apnoea-hypopnea index during rapid eye movement and non-rapid eye movement sleep in obstructive sleep apnoea J Int Med Res M 18831883 10.1177/147323000803600506

[26] Siddiqui F Walters AS Goldstein D Lahey M Desai H 7 3 2006 281 285 Half of patients with obstructive sleep apnea have a higher NREM AHI than REM AHI Sleep Med S 16564214 10.1016/j.sleep.2005.10.006

[27] Gay P, Weaver T, Loube D, Iber C, Positive Airway Pressure Task Force, Standards of Practice Committee, American Academy of Sleep Medicine (2006). Evaluation of positive airway pressure treatment for sleep related breathing disorders in adults. Sleep.

[28] Budhiraja R, Parthasarathy S, Drake CL, Roth T, Sharief I, Budhiraja P (2007). Early CPAP use identifies subsequent adherence to CPAP therapy. Sleep.

[29] Barbé R, Mayoralas LR, Duran J, Masa JF, Maimó A, Montserrat JM (2001). Treatment with continuous positive airway pressure is not effective in patients with sleep apnea but no daytime sleepiness. a randomized, controlled trial. Ann Intern Med.

[30] Russo-Magno P, O'Brien A, Panciera T, Rounds S (2001). Compliance with CPAP therapy in older men with obstructive sleep apnea. J Am Geriatr Soc.

[31] Weaver T, Grunstein RR (2008). Adherence to continuous positive airway pressure therapy: the challenge to effective treatment. Proc Am Thorac Soc.

